# The role of motivation factors in exergame interventions for fall prevention in older adults: A systematic review and meta-analysis

**DOI:** 10.3389/fneur.2022.903673

**Published:** 2022-08-05

**Authors:** Margot Buyle, Yujin Jung, Marousa Pavlou, Sergi Costafreda Gonzalez, Doris-Eva Bamiou

**Affiliations:** ^1^Psychological Sciences Research Institute and Institute of NeuroScience, Université Catholique de Louvain, Louvain-la-Neuve, Belgium; ^2^Audiology Department, Guy's and St Thomas' National Health Service Foundation Trust, London, United Kingdom; ^3^Faculty of Life Sciences and Medicine, Centre for Human and Applied Physiological Sciences, King's College London, London, United Kingdom; ^4^Division of Psychiatry, Faculty of Brain Sciences, University College London, London, United Kingdom; ^5^University College London Ear Institute, Faculty of Brain Sciences, University College London, London, United Kingdom; ^6^Biomedical Research Centre, National Institute for Health Research, London, United Kingdom

**Keywords:** falls, exergames, motivation, cognition, elderly, meta-analysis

## Abstract

Balance disorders and falls are common in the elderly population. Regular balance exercises are an evidence-based physical intervention to prevent falls in older adults, while patient motivation and adherence are important factors for intervention outcome. Exergames are a relatively new, alternative intervention for physical rehabilitation as they improve balance and strength in older adults. The aims of this systematic review and meta-analysis were to assess the (1) effect of motivation factors as per the Capability, Opportunity and Motivation model of Behavior change (COM-B) on the effectiveness of exergame interventions in healthy older adults, (2) effectiveness of exergames to improve balance in older healthy adults and, (3) impact of exergames on cognitive outcomes. Results show that motivation and capability components influence the general outcome of the exergame training. Motivational factors should thus be considered when setting-up an exergame intervention. Furthermore, exergame intervention appears to be a promising training method in comparison to traditional exercise training. However, exergame training in itself might not be sufficient to improve fall risk and cognitive performance.

## Introduction

Falls are common and a leading cause of disease and related disability in older adults (1–4). Changes in balance control due to advancing age can co-occur with age-related decline in cognition (2, 5–7). Impairment of executive functioning, in particular, is associated with increased fall risk and, poorer gait and balance performance (7–9). Regular exercises are recommended to prevent falls in older adults (10, 11) as the most effective intervention to reduce the incidence of falls (12).

Exergames provide a combination of “exercise” and “gaming” and have been increasingly used to improve balance and strength in older adults (13, 14). Exergames can offer computerized individual personalized exercises, progress reports, auditory and visual feedback, as well as additional motivational elements, like music and encouraging commentaries [see (15) for a review; (16)]. In addition, they can be practiced at home alone or in a group [(15) for a review].

Yang et al. (17) examined community older adults who played Kinect exergames for 5 weeks, and showed that exergames were more effective in terms of overall balance ability especially for the Functional Reach Test (FRT). Exergames were thus a good alternative for balance training, compared to the control intervention of conventional exercises [based on (18)] (17). Similarly, the Stanmore et al. randomized controlled trial showed that a 12-week exergames program targeting balance and strength in adults aged 55 years and older in the UK, reduced fear of falling, improved balance and was cost-effective in terms of fall prevention (19). Other studies have also reported exergames as effective rehabilitation tools for balance training, especially for the elderly population (20–22).

Exergames may also be beneficial for cognition (23–25), as well as for combined motor and cognitive functions (22, 26, 27). Combined physical and cognitive training interventions show larger effects on cognitive functions than either intervention on its own [(28–30) for reviews]. Exergaming has therefore become an alternative to other forms of computerized and non-computerized cognitive training (23).

Adherence to balance exercises and exergaming is essential for these to provide benefit. Older adults at risk of falling report that lack of motivation to exercise is a significant barrier, but they can be motivated to adhere to balance exercises if they perceive potential exercise benefits, such as prospects of independence and physical balance and gait improvement (31). Recent clinical trials include a range of motivational strategies within their design, such as supervision or remote feedback, prompts and memory aids, together with individualized goal setting and exercise prescription (32), to improve adherence. A longitudinal study of physical exercise performance by 18 older adults showed high levels of exercise adherence over 13 months, with key factors promoting adherence identified as appropriate exercise difficulty, social interaction (friendship), therapist attention, and exercise variety (33). Exergames are considered as an attractive alternative to conventional leaflet-based non-supervised exercises, in that they may promote motivation by providing more enjoyable exercises and a social context, in addition to automatic feedback.

The Capability, Opportunity and Motivation (COM-B) psychological model of behavior change has been used to identify factors related to successful behavioral change and specify targets to help modify health-related behavior (34) as well as in studies assessing gamification of mobile health interventions (35, 36), and motivational interventions for older adults who fall (37). This theoretical framework is consistent, parsimonious and evidence-based (38).

The purpose of this systematic review was to: (1) examine if exergames are effective in improving balance and preventing falls in older adults; (2) investigate the effects of exergames on cognitive outcomes; and (3) explore if motivation factors, identified by applying the COM-B behavior model and behavior change wheel, have an impact on the effectiveness of exergame interventions in older adults.

## Methods

This systematic review and meta-analysis were performed in accordance with PRISMA guidelines (39).

### Eligibility criteria

Only full-text, peer reviewed, randomized control trials published in English were included in this study. Inclusion criteria were: (a) participants had to be at least 65 years of age (no higher age limit), (b) participants were healthy community-dwelling older adults without a diagnosis of neurological conditions affecting balance such as Parkinson's disease and stroke, mental or memories problems, (c) exergame interventions aimed at falls prevention and/or improvement of balance in older adults, (d) at least one control group who either received a conventional exercise intervention (e.g., fall prevention exercise, ball exercise, habitual exercise, traditional Tai-Chi) or no intervention, and had a pre-post intervention assessment. The primary collected outcomes of this systematic review were the effectiveness of the exergames to improve balance and/or prevent falls. Appropriate gait, postural balance and cognitive function measures were used to evaluate efficacy by comparing pre-post outcome measure data for the intervention and control groups. Exclusion criteria were as follows: (a) participants with history of serious falls, chronic or acute orthopedic, mental health, balance, cardiac and/or visual impairment, (b) participants in hospital, care home, or institutional care, and (c) studies with no control group.

### Search strategy

Two searches were conducted using three electronic databases (Pubmed, Scopus and Web of Science) to identify the highest number of eligible articles possible. These were searched by using the same keywords for each database and with searches limited to manuscripts printed in English. Each electronic database was searched from January 2000 to until May 2021.

In this systematic review, the Boolean operators AND or OR were used in all database searches to combine search keywords. The first search included following keywords: “older or old or elderly or aged or ag^*^ing or adult^*^ or senior^*^”, “fall^*^ or balance or vestibular OR train^*^ OR exercis^*^”, “exergam^*^ or exer-gam^*^ or exergaming or virtual” and, “intervention or prevention”. The second search consisted of these keywords: “older or old or elderly or aged or aging or adult^*^ or senior”, “fall^*^ or balance or vestibular or train^*^ or exercis^*^”, “exergam^*^ or exer-gam^*^ or exergaming or virtual”, and, “fall and intervention or prevention”.

### Study strategy and data extraction

**Figure 1:**
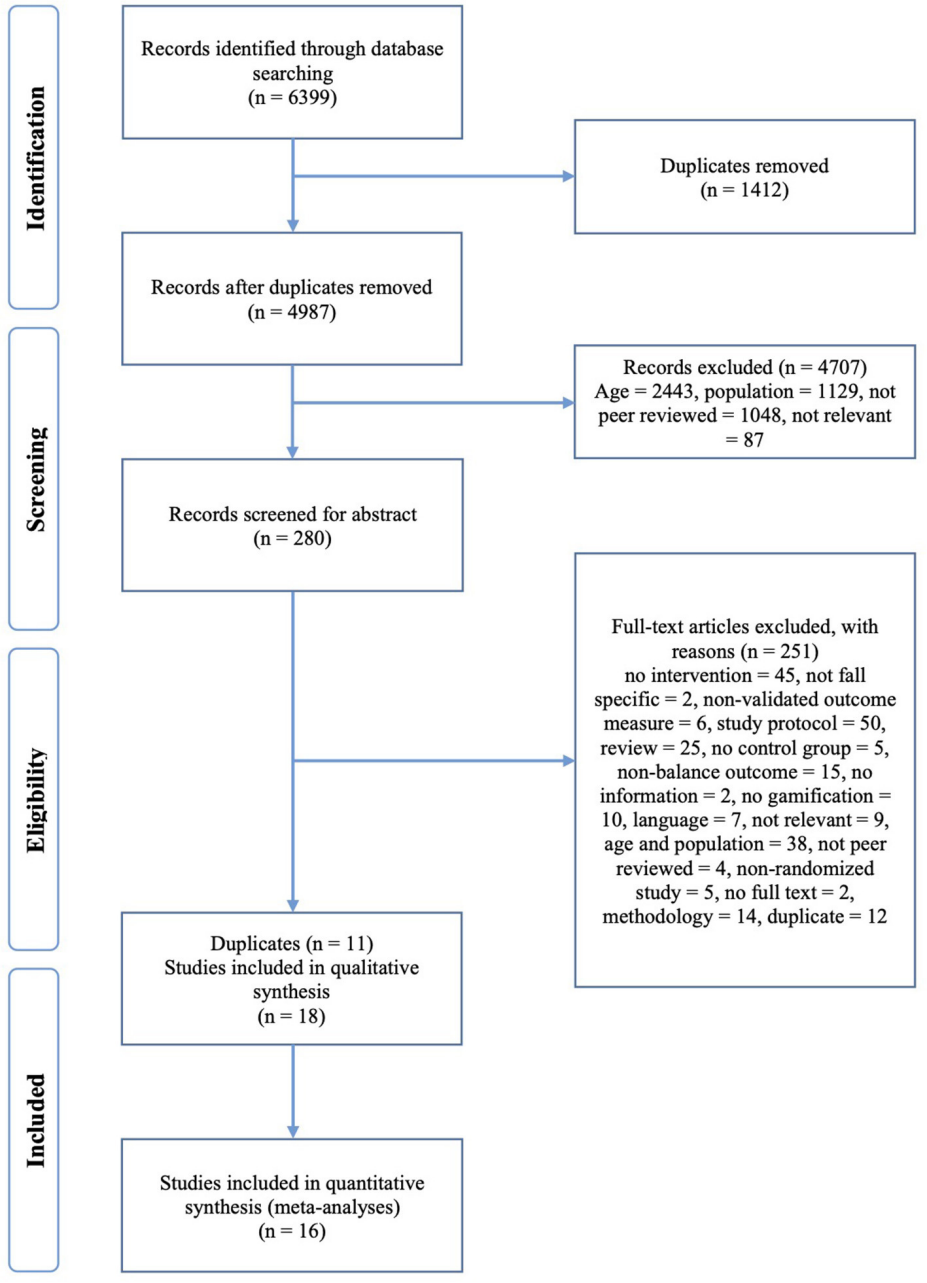
PRISMA flowchart of the article selection processes.

A first stage search identified 6,399 possible related papers in total. Descriptive data and study outcome details were synthesized and tabulated. First, 1,412 duplicates were removed, resulting in 4,987 remaining records, respectively. Potential relevant papers were then screened by two reviewers according to specified inclusion criteria, which led to 280 remaining records. These records were screened by reading the abstract resulting in the removal of 251 records for not meeting the inclusion criteria. Exclusion reasons are listed in the PRISMA diagram (see Figure 1). Finally, 29 studies were identified according to the eligibility criteria for the two searches, and after removing duplicates a total of eighteen studies were defined for the systematic review. Sixteen out of eighteen studies were found to be eligible for meta-analyses.

Since the aim of this review was to explore the effectiveness of exergames, and which motivation factors of exergames have an impact on improving balance and preventing falls, interventions were identified and analyzed using the COM-B model. The COM-B model posits that the interaction between Capability, Opportunity and Motivation (COM) causes the changes in Behavior (B). Capability is the “individual's psychological and physical capacity to engage in the activity concerned”. Opportunity includes the “factors that lie outside the individual that make the behavior possible or prompt it”. Motivation includes Reflective Motivation (evaluations, intentions and plans) and Automatic Motivation (emotions and impulses arising from learning and innate dispositions) (34). Data information extracted from included studies consisted of the following: author, year, type of the exergame technologies used, motivational factors as per COM-B, participant information, intervention setting and outcomes (see Supplementary Table 1; Table 2). Figure 1 presents the screening and selection process for the studies included in this systematic review.

### Data analysis

Raw data was tabulated using Microsoft excel. IBM SPSS Statistics version 26 for Mac was used for data analysis. Meta-analysis was performed using the Cochrane Review Manager software (RevMan 5.4). Studies that had similar outcome measures were included in a random effects model. Meta-analysis effect sizes (reported as Standard Mean Differences = SMD) were calculated for pre-post intervention comparisons within each group. Interpretation of effect size values (SMD) was as follows: 0.20–0.49, 0.50–0.79, and > 0.80 indicated a small, medium, and large effect size, respectively (40). Forest plots were used to summarize outcomes for each meta-analysis. Second, the components of the exergames interventions were classified following the COM-B model into those addressing capability, opportunity and/or motivation, following the framework of (34). *I*^2^ measures of heterogeneity were used with values of 75, 50, and 25% indicating high, medium and low heterogeneity (41).

## Results

Eighteen studies in total met the inclusion criteria and were assessed in this systematic review. Risk of bias for each study was assessed using the AXIS critical appraisal tool for cross-sectional studies (42). Abstracts and full articles were reviewed for inclusion criteria by MB, and double-rated by a second reviewer (D-EB) with disparities in opinion resolved through discussion or review by a third rater (MP). Among these, sixteen studies provided quantitative data suitable for various quantitative meta-analyses. Supplementary Table 1 summarizes the characteristics of the eighteen studies, all of which were published between 2013 and 2021.

### Study characteristics

All included studies aimed to evaluate the effect of exergame training on physical functions such as balance and muscle strength, and/or cognitive functions (see Supplementary Table 1 for details). Eleven of these (43–53) evaluated the effectiveness of exergames on balance performance. Eight studies (45, 46, 50, 53–57) evaluated the effectiveness of exergames on cognitive functions. All studies assessed participants at baseline and at the end of the intervention. Only two studies (45, 58) conducted long-term monitoring for fall frequency and adverse events for 6–12 months' post-intervention.

### Participants characteristics

A total of 909 participants were included in the eighteen studies. The samples included healthy community-dwelling participants age 65 years or older, with a mean age of 77.62 ± 5.78 years old, who were able to walk independently and were capable of conducting the exergames either without supervision after training, or with supervision depending on the study. For each study, the number of participants ranged from 20 to 153. The percentage of women was 60.64% (female-to-male ratio of approximately 6:4). Two studies (51, 59) recruited only male participants, while all other studies included both sexes. Two studies (53, 60) did not report sex percentage.

### Outcome measures

All studies assessed the participants at baseline before starting the intervention to compare with post-intervention. The outcome measures were all validated measures commonly used in clinical practice. A diversity of outcome measures was used to evaluate the effect of the exergame intervention vs. the control group (see Table 1).

**Table 1:** Outcome measures.

**References**		**Outcome measures**				
	**Physical function**	**Balance**	**Strength**	**Cognitive function**	**Questionnaire**	**Others**
Adcock et al. (54)	✓	✓		✓		MRI
Chao et al. (43)	✓	✓			✓	
Chen et al. (44)		✓	✓			
Cho et al. (60)		✓				
Eggenberger et al. (58)	✓				✓	
Eggenberger et al. (57)	✓			✓	✓	
Gschwind et al. (45)	✓			✓	✓	
Gschwind et al. (46)	✓			✓		
Katajapuu et al. (47)	✓	✓				
Lee et al. (48)	✓	✓				
Li et al. (56)	✓	✓		✓		
Park et al. (49)		✓	✓			
Park and Yim (55)		✓	✓	✓		
Phirom et al. (50)	✓			✓		
Sadeghi et al. (59)	✓					
Sadeghi et al. (51)		✓	✓			
Sato et al. (52)	✓	✓	✓			
Schoene et al. (53)	✓	✓		✓	✓	
Total	13	12	5	8	5	1

#### Balance outcome measures

The most frequently used balance outcome measures in this review were the: Timed Up and Go test (TUG), Berg Balance Scale (BBS), Functional Reach Test (FRT), Physiological Profile Assessment (PPA) and Sit to Stand test (STS).

The Timed Up and Go test (TUG) is a dynamic balance and physical function measure assessed in nine studies (43–46, 48–51, 53). The TUG score is measured in seconds with a cut-off value of 14 s (61).

The Berg Balance Scale (BBS) is also a balance measure implemented in five studies (43, 44, 47, 48, 52). BBS scores evaluate a set of 14 tasks related to balance. Each task is rated on a scale of 0–4. Total final scores compute a sum of all the 14 tasks and scores range from 0 to 56 (excellent balance). A cut-off score of 45 has been determined to identify a greater risk of falling (62).

The Functional Reach Test (FRT) assesses static balance and was used in three studies (44, 48, 52). The test measures the maximal forward distance a participant can reach beyond the length of the arms in standing and is measured in cm against a wall at shoulder height. A reach distance of ≥ 25 cm indicates low falls risk (63).

The Physiological Profile Assessment (PPA) estimates fall risk based on five sensory motor tasks and was used in four studies (45, 46, 50, 53). The PPA consists of five tests that provide scores in six levels: below −1 very low, −1 to 0 low, 0–1 mild, 1–2 moderate, 2–3 high falls risk.

The Sit to Stand test (STS), a muscle strength test, is measured in seconds and consists of five repetitions. This was included in two studies (46, 48). Normative scores are defined as a cut-off value of 12 s (64, 65).

#### Cognitive outcome measures

Eight studies (45, 46, 50, 53–57) assessed cognitive function with outcome measure tools varying across studies: Attention Network Test (ANT), Digit Span Backward test (DSB), Montreal Cognitive Assessment (MoCA) and Trial Making Test (TMT).

Three studies (45, 46, 56) assessed the Attention Network Test (ANT). During the ANT participants need to determine whether a central arrow points to the left or the right. Processing efficiency within three attentional networks (alerting, orienting and executive attention) is quantified. Influence of altering cues, spatial cues and flankers on reaction times is measured (66).

Another cognitive measure included in two studies (45, 46) was the Digit Span Backward test (DSB), which measures working memory and requires participants to repeat number sequences with increasing length in reverse order (67).

The Montreal Cognitive Assessment (MoCA), a clinical tool for screening mild cognitive impairment, was used in three studies (50, 55, 57). The MoCA examines eight cognitive domains: attention and concentration, executive functions, memory, language, visuo-constructional skills, conceptual thinking, calculations, and orientation. The maximum score is 30 points and a score of ≥26 is considered normal for older adults (68–70).

The Trial Making Test (TMT), examines psychomotor speed and executive functioning, and was included in four studies (45, 53, 54, 57). During the first part of the test (TMT A) participants are asked to connect numbers from 1 to 25 in ascending order as fast as possible. In the second part (TMT B), participants must connect number and letters in alternating ascending numerical and alphabetical order, as fast as possible. Time is recorded in seconds and errors are also counted (71–73).

### Technology of intervention

A variety of different technologies and exergames were used as an intervention method to prevent falls and/or improve balance (see Supplementary Table 1 for details). The exergames were carried out either at home or at a place with the right setting (i.e., welfare center, health promotion hospital, village health club, senior center, clinic, research institute, etc.). Only one study (46) did a comparison of two exergame interventions.

All studies included a control group who received conventional exercises (e.g., fall prevention exercise, ball exercise, habitual exercise, traditional Tai-Chi) or no intervention. The exergame intervention duration varied across seventeen out of eighteen studies ranging from 4 to 24 weeks (Mean ± SD = 9.76 ± 5.33); one study (52) did not report duration. The duration and frequency of each exergame session ranged from 17.50 to 60 min (Mean ± SD = 43.19 ± 13.66) and 2–3 times weekly (Mean ± SD = 2.69 ± 0.44), respectively. Two studies (45, 46) did not report the frequency of the exergame intervention and one study (52) did not report intervention duration. The total dosage of exergame playing for each intervention ranged from 240 to 2,880 min (Mean ± SD = 1,063.33 ± 801.24).

In nine studies, the control group did not receive any training intervention but was asked to perform daily activities as usual (45, 46, 51–54, 56, 59, 60). Four (44, 49, 55, 57) studies used a control exercise program [e.g., traditional Tai-Chi (74) exercises; ball game proposed by (75); conventional balance exercises], and two studies (47, 58) used two different intervention arms: physiotherapy exercises and no intervention; treadmill walking with memory vs. treadmill walking. Three studies (43, 48, 50) used fall prevention/health education as the control intervention.

### Gamification design

Most studies used commercially available and popular gaming technologies, which are not tailored for older people but rather designed to entertain young people. Seven studies used the X-box Kinect games (44, 46, 47, 50–52, 59), that can be controlled by gestures and spoken commands with no need of a game controller (76). Four studies used the Nintendo Wii Fit games (43, 48, 49, 60). These games use a hand controller and/or a Wii Fit board to sense rotational motion and acceleration and/or body movement information. Three studies used dance video games (like for example the Dance Dance Revolution) (53, 57, 58) which often use a dance mat with pressure sensors for sensing steps (53). Of the remaining studies one study (55) used a kayak exergame, one study a Virtual Reality motion game (56), one study the iStoppFalls gamification design (45), and a last study used the Active@Home exergame (54) (see Supplementary Table 1 for more detailed information).

### Meta-analyses

Nine studies were included in a separate meta-analysis for balance and muscle strength. Seven studies (54–60) evaluated balance using other tests, one study (51) showed outlier outcomes and therefore probably implausible findings (i.e., effect size of 5), and one study (47) did not include sufficient data such as SD and post outcomes, and were excluded from the meta-analysis. Eight studies included cognitive measures, but only five studies were included in the meta-analysis for cognitive function, as two studies (54, 56) did not provide sufficient data and outcomes could therefore not be evaluated.

### COM-B model

In this systematic review, the eighteen identified studies were analyzed to evaluate the effect of motivational factors on the results of the exergame interventions using the COM-B model. Reflective and automatic **motivation** components were incorporated within intervention in all studies, mostly consisting of auditory and visual feedback.

Ten studies' interventions (43, 45–48, 53, 54, 56–58) included a physical and social **opportunity** component of the COM-B model. Four studies were conducted at home without supervision (45, 46, 53, 54) and provided support by phone call. Two studies gave a shopping voucher when completing 90% of all visits during the whole intervention period, or offered some money in compensation per hour (47, 56). One study scheduled a reminder by tablet, and provided on-site monitoring and support by an assistant (48). Three studies provided supervision (43, 57, 58).

The interventions of eleven studies were associated with both the physical and psychological **capability** component of the COM-B model. The interventions of seven studies (45, 51, 53, 55, 57–59) involved physical capability by receiving extra exercises and sessions, offering additional home visits and individualized exercises. In terms of psychological capability, interventions consisted of education and providing a manual in four studies (43, 46, 48, 54).

**Figure 2:**
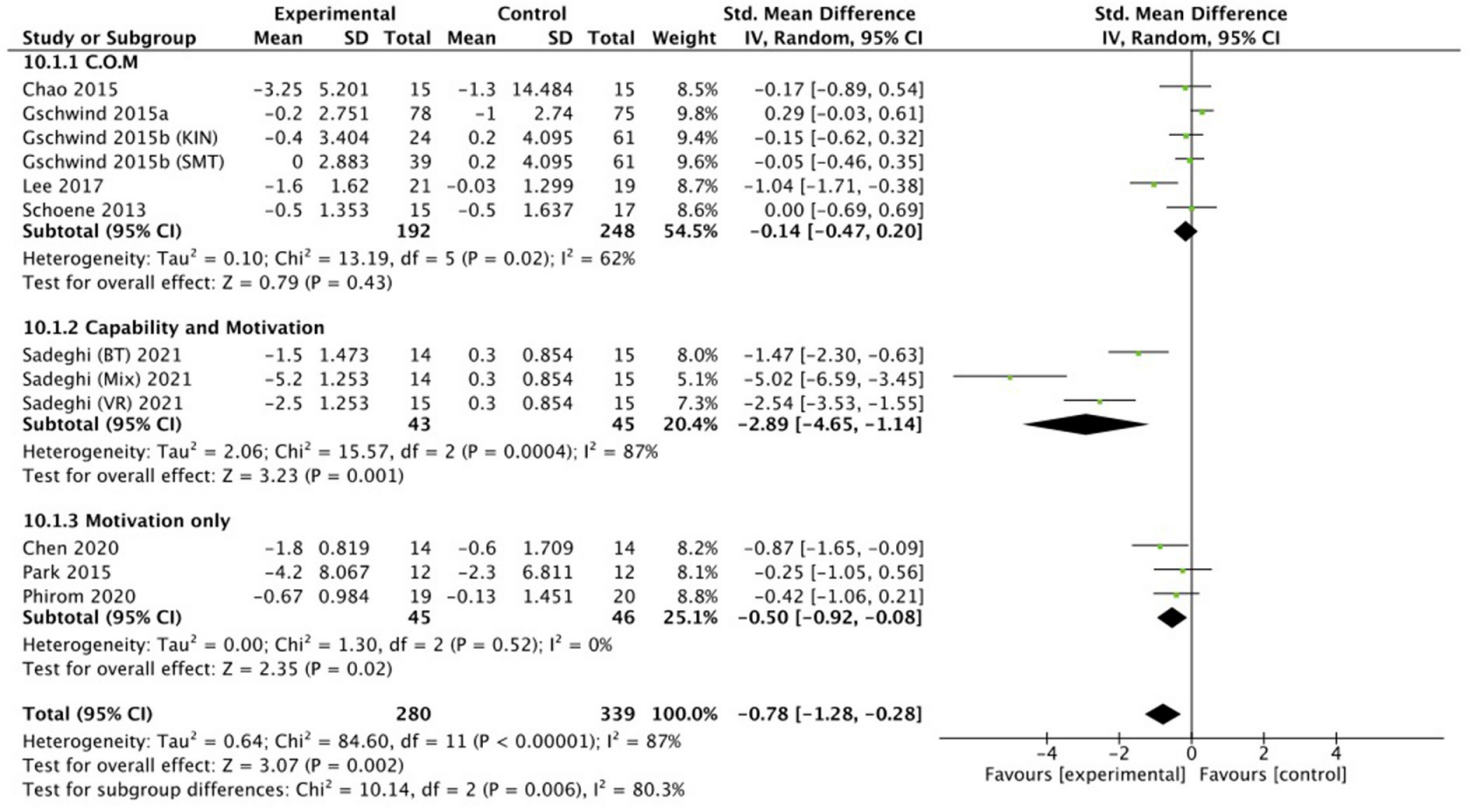
Illustration of the forest plot for evaluating interventions involving capability, opportunity and motivation vs. capability and motivation vs. motivation only for effectiveness of intervention on measures of TUG balance outcomes.

Eight studies (43, 45, 46, 48, 53, 54, 57, 58) evaluated all three components of the COM-B model and three studies (51, 55, 59) evaluated two components of the COM-B model, capability and motivation. Two studies (47, 56) evaluated two other components of the COM-B model, namely opportunity and motivation. Five studies (44, 49, 50, 52, 60) evaluated only the motivation component (see Table 2 for more details). As one aim of this study was to investigate which motivational elements may impact the effectiveness of the exergame interventions on balance outcomes, the meta-analysis was conducted including all nine studies that had TUG scores as common value. Therefore, five studies acted on all three components of the COM-B model (43, 45, 46, 48, 53), one study acted on two components (capability and motivation: (51), and three studies acted on motivation only (44, 49, 50). Meta-analysis results indicated that all three components displayed small and not significant effects (SMD = −0.14, 95% CI = −0.47 to 0.20, *I*^2^ = 62%, Chi^2^ = 13.19, *p* = 0.43). Capability and motivation components indicated a large and significant effect (SMD = −2.89, 95% CI = −4.65 to −1.14, *I*^2^ = 87%, Chi^2^ = 15.57, *p* = 0.001). Motivation only indicated a medium significant effect (SMD = −0.50, 95% CI = −0.92 to −0.08, *I*^2^ = 0%, Chi^2^ = 1.30, *p* = 0.02), see Figure 2.

**Table 2:** Elements of motivation identified using the COM-B model.

**References**	**COM-B model components**		
	**Capability**	**Opportunity**	**Motivation**
Adcock et al. (54)	Spoken and written instructions.	• Exercise at home. • Phone support available.	Providing visual and auditory feedback.
Chao et al. (43)	Health education.	• Supervision provided. • Participants supported to encourage each other. • 4-point walker was provided for safety.	Automatically increased the levels.
Chen et al. (44)			Received real time feedback. Automatic measurement of levels. Provided accuracy movements.
Cho et al. (60)			Participants could see their feedback.
Eggenberger et al. (58)	Individualized exercises.	• Supervision provided. • Rope provided to confirm the safety.	Participants could see their results. Provided accuracy movements.
Eggenberger et al. (57)	Individualized exercises.	• Supervision provided. • Rope provided to maintain balance.	• Participants could see their results. • Provided accuracy movements. • Automatically increased the levels.
Gschwind et al. (45)	Home visit to ensure training.	• Exercise at home. • Phone support available and home visit if required. • Reminders through the tablet and assistant.	Participants could see their results and get feedback.
Gschwind et al. (46)	Provided manuals and instructions.	Exercise at home.	• Received phone calls monthly. • Participants could see their results.
Katajapuu et al. (47)		• Given voucher of $70. • Supervision provided to confirm the safety.	Enjoyment for exercise.
Lee et al. (48)	Fall prevention education.	Assistants support to encourage participants.	• Enjoyment for exercise. • Providing visual and auditory feedback.
Li et al. (56)		Given voucher of $10 per hour.	Enjoyment for exercise. Providing visual and auditory feedback. Participants could select the levels.
Park et al. (49)			• Enjoyment for exercise. • Providing visual and auditory feedback.
Park and Yim (55)	Extra conventional exercises for 30 min.		Enjoyment for exercise.
Phirom et al. (50)			Enjoyment for the targets. Automatically increased the levels.
Sadeghi et al. (59)	Individualized exercises.		• Automatically increased the levels. • Participants could see their results and get feedback.
Sadeghi et al. (51)	Individualized exercises.		Increment of levels/repetitions.
Sato et al. (52)			• Enjoyment for exercise. • Automatically increased the levels.
Schoene et al. (53)	• Extra session (CSRT) to enhance movement speed. • Manual with step by step instructions.	• Exercise at home. • Phone support available.	• Enjoyment for exercise to target. • Participants could see their feedback and select the levels.

#### Meta-analyses of balance outcome measures

**Figure 3:**
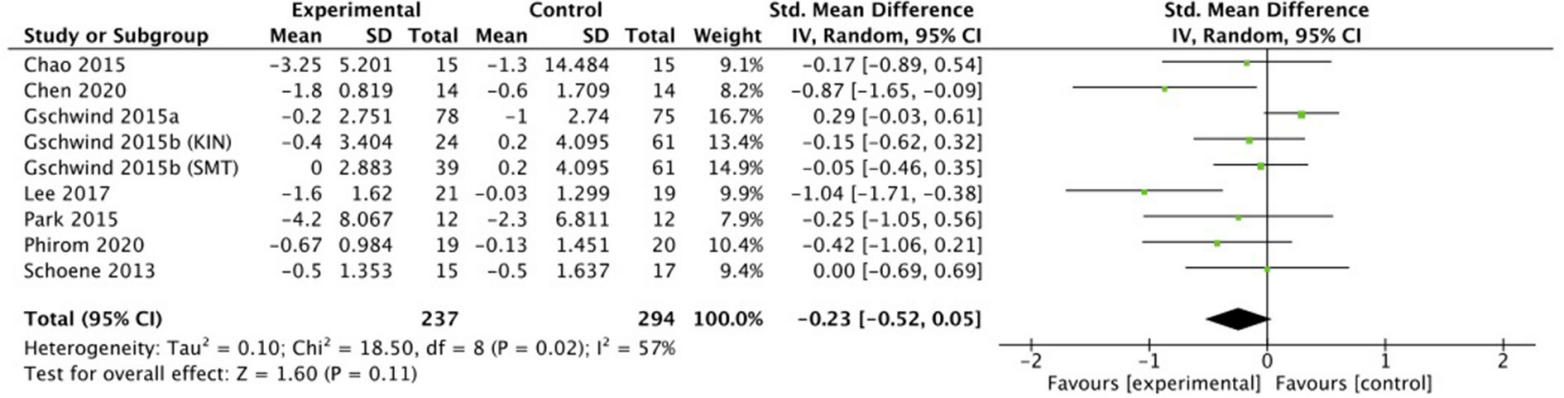
Random effects meta-analysis forest plot for effectiveness of intervention on measures of TUG balance outcomes.

**Figure 4:**
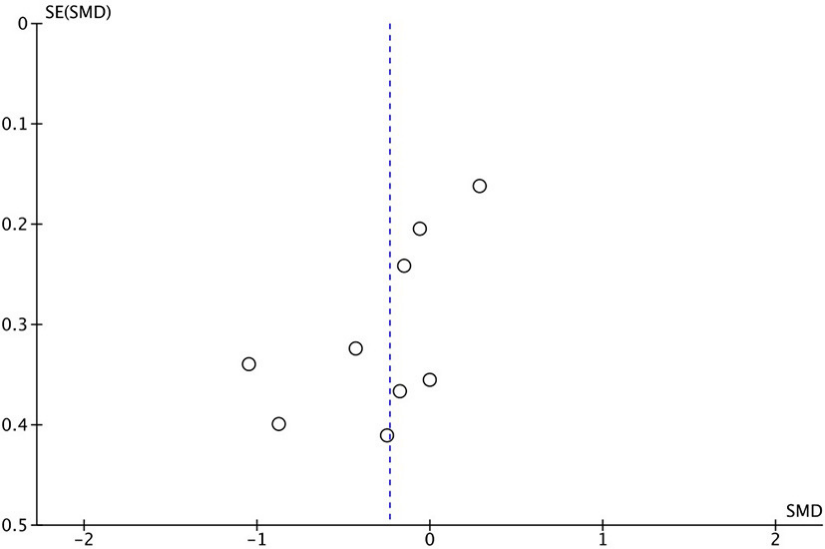
Funnel plot of comparison: TUG.

Eight studies (*n* = 531) were included in a meta-analysis for the effectiveness of exergame intervention on balance using the **TUG**. A total of 237 participants were included in the exergame intervention group whereas 294 participants were included in a control group. However, note that one study (46) included two exergame intervention groups and one control group. A small effect size was observed: the exergaming intervention did not significantly reduce the TUG time (SMD = −0.23, 95% CI = −0.52 to 0.05, *I*^2^ = 57%, Chi^2^ = 18.5, *p* = 0.11), see Figures 3, 4.

**Figure 5:**
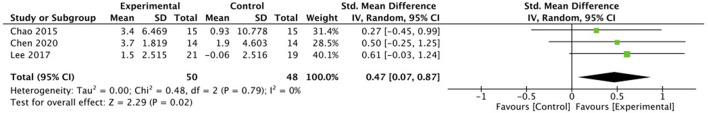
Random effects meta-analysis forest plot for effectiveness of intervention on measures of BBS balance outcomes.

Five studies conducted the effect of dynamic balance using the **BBS**. Only three studies (*n* = 98) were included in a meta-analysis for the effectiveness of exercise intervention on balance using the BBS test, as two studies did not include sufficient information (47, 52). The total number of participants in the exergame intervention group was 50 and 48 for the control group. The exergaming intervention significantly improved the BBS score as indicated by a medium effect (SMD = 0.47, 95% CI = 0.07 to 0.87, *I*^2^ = 0%, Chi^2^ = 0.48, *p* = 0.02), see Figure 5.

**Figure 6:**
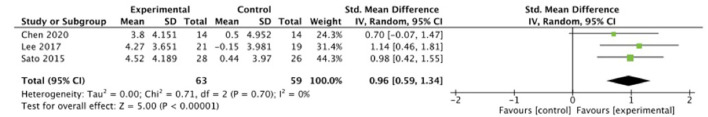
Random effects meta-analysis forest plot for effectiveness of intervention on measures of FRT balance outcomes.

Three studies (*n* = 122) conducted the effect of static balance for the **FRT** test. The number of participants in the exercise intervention group was 63 and 59 in the control group. A large effect size was observed, where the exergaming intervention was significantly more efficacious than the control intervention for the FRT test (SMD = 0.96, 95% CI = 0.59 to 1.34, *I*^2^ = 0%, Chi^2^ = 0.71, *p* < 0.00001), see Figure 6.

**Figure 7:**
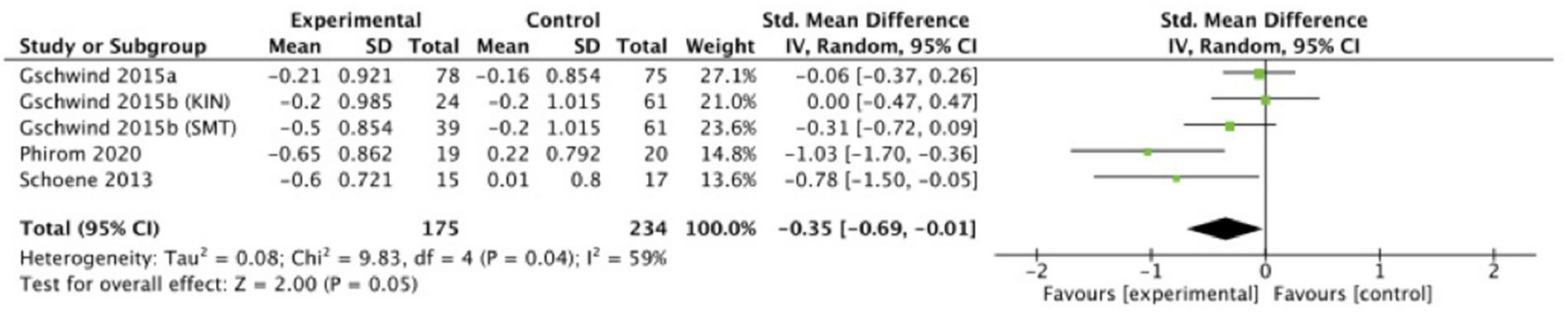
Random effects meta-analysis forest plot for effectiveness of intervention on measures of PPA physical outcomes.

Four studies (*n* = 409) were included in a meta-analysis for the effectiveness of exercise intervention on falls risk based on the **PPA**. Figure 5 illustrates the effect of physiological functions related to postural stability. A total of 175 participants were included in the exergame intervention group whereas 234 participants were included in a control group. Small significant effects of exergame intervention were observed for postural stability (SMD = −0.35, 95% CI = −0.69 to −0.01, *I*^2^ = 59%, Chi^2^ = 9.83, *p* = 0.05), see Figure 7.

**Figure 8:**
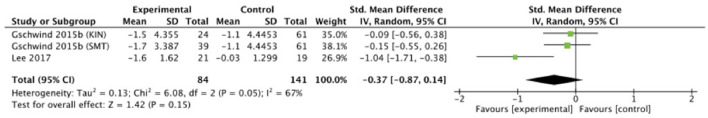
Random effects meta-analysis forest plot for effectiveness of intervention on measures of STS strength outcomes.

Two studies (*n* = 370) were included in a meta-analysis for the effectiveness of exercise intervention on muscle strength. Figure 6 illustrates the effect of muscle strength for the **STS**. Only two studies conducted STS, however, one study (46) assessed two different exercise interventions. Therefore, three data comparisons were pooled. The exercise intervention group consisted of 84 participants and the control group consisted of 141 participants. Analysis of the three data sets indicated that there was a small, though not significant, effect of the exergame intervention compared with the control group (SMD = −0.37, 95% CI = −0.87 to 0.14, *I*^2^ = 67%, Chi^2^ = 6.08, *p* = 0.15), see Figure 8.

#### Meta-analyses of cognitive outcome measures

**Figure 9:**
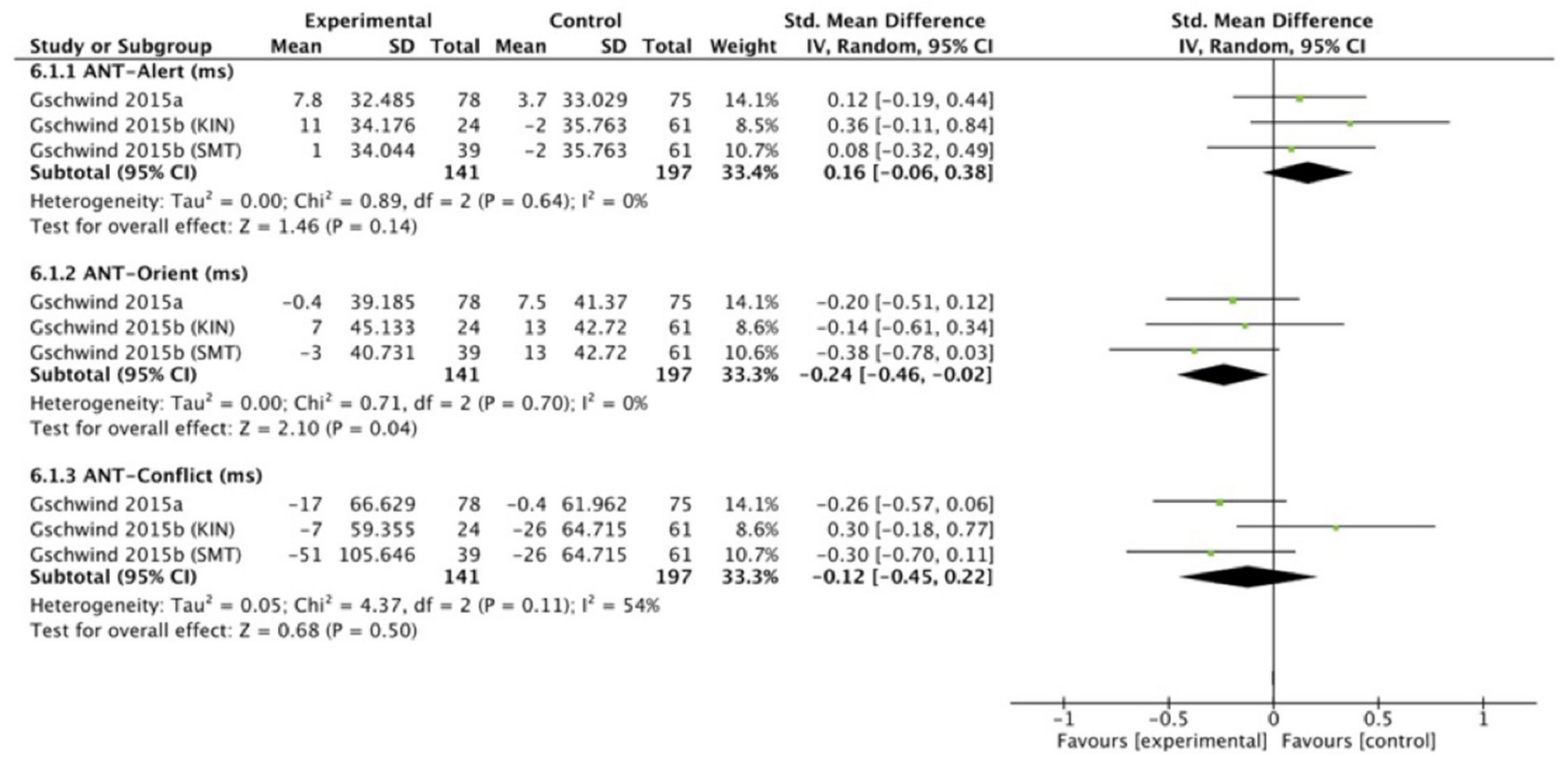
Random effects meta-analysis forest plot for effectiveness of intervention on measures of ANT cognitive performance outcomes.

A variety of outcome measures were used across studies to assess cognitive function. It was not possible to conduct a meta-analysis with the eight studies that evaluated cognitive function, because they evaluated different cognitive tests. Five studies were therefore included in the meta-analysis. Two out of the five studies (*n* = 277) were included in a separate meta-analysis using two matching outcomes, namely the **ANT**. The outcomes were pooled for three attentional components; alerting, orienting and executive control. The total number of participants of the exercise intervention group was 141 and 197 in the control group. For all three components, small effects were observed (SMD = 0.16, 95% CI = −0.06 to 0.38, *I*^2^ = 0%, Chi^2^ =.89, *p* = 0.14 for alerting; SMD = −0.24, 95% CI = −0.46 to −0.02, *I*^2^ = 0%, Chi^2^ = 0.71, *p* = 0.04 for orienting; SMD = −0.12, 95% CI = −0.45 to 0.22, *I*^2^ = 54%, Chi^2^ = 4.37, *p* = 0.5 for executive control). Figure 9 illustrates the effect of cognitive performance for the ANT.

**Figure 10:**
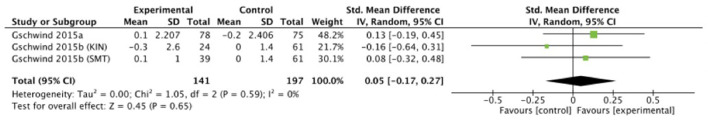
Random effects meta-analysis forest plot for effectiveness of intervention on measures of DSB cognitive performance outcomes.

The same two studies were included in a meta-analysis to illustrate the effect of cognitive performance for the **DSB**, which indicated a very small, non-significant, effect of exergame intervention in comparison to control intervention (SMD = 0.05, 95% CI = −0.17 to 0.27, *I*^2^ = 0%, Chi^2^ = 1.05, *p* = 0.65), see Figure 10.

**Figure 11:**
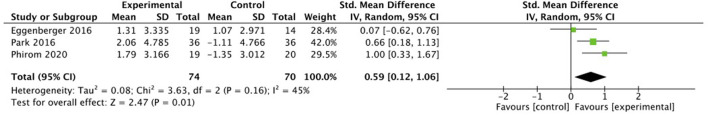
Random effects meta-analysis forest plot for effectiveness of intervention on measures of MoCA cognitive performance outcomes.

Another three out of the eight studies (*n* = 144) were included in a meta-analysis for the effectiveness of exercise intervention on cognitive performance from the **MoCA**. The exercise intervention group consisted of 74 participants and the control group consisted of 70 participants. Exergame intervention showed a medium effect for MoCA cognitive performance compared with the control group, which was significant (SMD = 0.59, 95% CI = 1.12 to 1.06, *I*^2^ = 45 %, Chi^2^ = 3.63, *p* = 0.01), see Figure 11.

**Figure 12:**
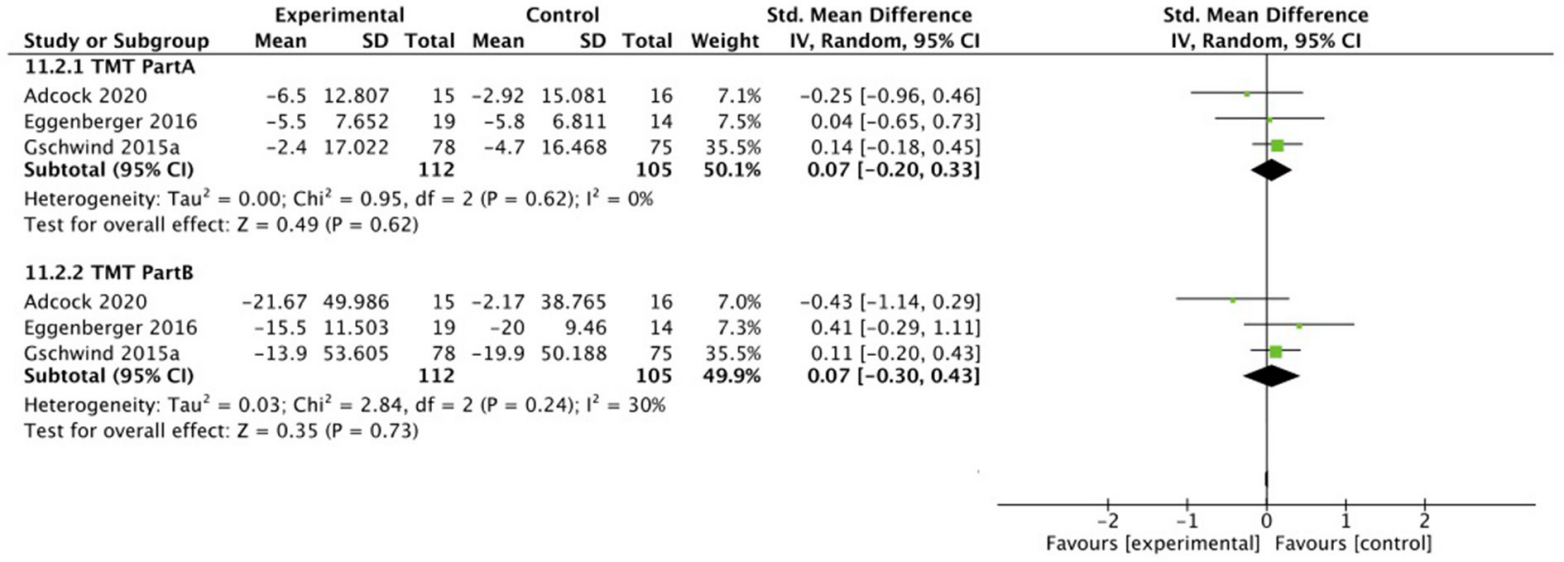
Random effects meta-analysis forest plot for effectiveness of intervention on measures of TMT cognitive performance outcomes.

Four out of the eight studies mentioned above conducted the **TMT** to assess cognitive function. One study (53) did not include sufficient data and used different outcome values. Therefore, only three studies (*n* = 271) were included (45, 54, 57) in a meta-analysis evaluating the effectiveness of intervention on TMT scores. The TMT test consists of two parts, part A and B. The outcomes were pooled from part A and B, respectively. The number of participants of the exercise intervention group was 112 and 105 in the control group. For both TMT part A and part B, small although not significant effects for exergame intervention were observed compared with control intervention (SMD = 0.07, 95% CI = −0.20 to 0.33, *I*^2^ = 0%, Chi^2^ = 0.95, *p* = 0.62 for TMT part A; SMD = 0.07, 95% CI = −0.30 to 0.43, *I*^2^ = 30%, Chi^2^ = 2.84, *p* = 0.73 for TMT part B), see Figure 12.

## Discussion

To our knowledge, this is the first systematic review to evaluate the role of motivation during exergames and how it impacts the effectiveness of exergames on improving balance, cognition and preventing falls in healthy older adults by applying the COM-B model.

### COM-B model meta-analysis

Meta-analysis results indicated that, overall, motivational components might impact the effectiveness of exergames on improving TUG scores when motivation alone, or capability and motivation are considered: A large significant effect was observed when capability and motivation components are considered during intervention. Including motivational factors alone resulted in a medium significant effect on the TUG outcome. Only Sadeghi et al. (51) included both components by providing individualized exercises, and by increasing the levels/repetitions. Three studies (44, 49, 50) included the motivation component in terms of providing visual and auditory feedback, enjoyment for the exercises, and by automatically increasing the levels. The impact of these factors have been considered before: healthy adults who performed exergame exercises with provision of feedback, for example, reported significantly higher enjoyment than adults performing the exercises in a conventional manner, with similar dynamic balance benefits in the two groups (77). Meekes and Stanmore (78) conducted interviews on older adults who performed 6 weeks of exergames (OTAGO and Fame provided on Microsoft Kinect) to improve their balance, in order to define factors that motivate. Enjoyment was one of the main motivational factors. Although feedback was provided by the games both during and after the game, participants stated that they wished for additional progress feedback provided by the physiotherapist (78).

van Het Reve et al. (79) found that tablet-based balance exercises together with motivational elements led to better exercise adherence and better gait outcomes when dual-tasking, compared to a brochure-based intervention. Inclusion of individual vs. social motivation strategies did not significantly impact on the gait parameter outcome measures, but exercise adherence was marginally better. Notably, active exercise performers were significantly more in the social vs. the individual motivation group (79). Silveira et al. similarly showed better (but not significantly) exercise adherence and lower attrition in the social vs. the individual motivation group, highlighting the importance of social interaction as a motivational factor for exercise performance (80). Li et al. on the other hand found that social interaction significantly affected the changes of extrinsic motivation over time, while competitive information affected intrinsic motivation significantly (81). Two out of the three studies in this meta-analysis were performed in an exercise group, so social motivation could have possibly contributed.

Dockx et al. showed that attitudes toward Virtual Reality exergames can positively change following exposure to it (82). Older people became enthusiastic about it, and showed improved health and physical functioning. In line with this, Subramanian et al. found that older adults were more motivated by the perceived health effects (both physical and cognitive) and the joy of playing, than by the in-game rewards (83). Game enjoyment was reported for exergames [see (76) for a review], and all studies included in the review of van Diest et al. showed that exergame intervention was more appealing than traditional exercises (13).

Hughes et al. (37) conducted a meta-analysis to assess how effective the different methods employed to promote exercise adherence are for older, non-neurological adults at risk of falls. The included studies were evaluated using the COM-B model to define the domains of the interventions. All of the studies included motivation for exercise, and some included opportunity, capability, or both. Studies using exergames, telecommunication, self-efficacy targeting, and integration of exercise into daily activity that provided good quality data were entered into a meta-analysis and showed significantly better adherence in the intervention group vs. the control indicating that motivational strategies are important for exercise adherence (37).

Overall, motivation is one of the most important factors to keep participants' adherence. Increasing enjoyment in exergames can be obtained via providing feedback and rewards, self-identification with the game characters (84, 85), and enjoyment (86). Several factors like self-determination, motivational feedback, competition and coordination, social interaction, and situational interest are active approaches. Moreover, personality-based tailoring is being explored to better satisfy individual and group differences [see (84) for a review].

Nevertheless, caution should be taken while interpreting the results of this study's meta-analysis, as the sample size was lower compared to the analysis with studies including all three components. When all three components of the COM-B model were included, a small, non-significant effect was seen. It is noteworthy that all measurements in the study of Lee et al. (48) significantly improved in the exergame intervention group. The other three studies also reported that there was an overall improvement in functions and abilities (45, 46, 53). The major difference lies in the fact that the three studies conducted the exergames at home without supervision, while the study of Lee et al. (48) was undertaken in a clinical setting with a supervisor.

The opportunity component of the COM-B model can be divided in physical opportunity and social opportunity. Three studies were home-based (physical opportunity) and provided phone support or tablet support (social opportunity, although not fully adapted) (45, 46, 53). Lee et al. (48) adopted fully social opportunity since assistants consistently monitored participants on-site to handle procedures and assure safety, during the whole program. The assistants also explained how to play the games, and encouraged the participants to actively participate (48). Although opportunity—combined with capability and motivation—did not show a significant impact on the effectiveness of exergames, the presence of a physiotherapist or supervision might have an influence too, as suggested by previously stated literature, and the fact that exercises were performed in classes in 2:3 studies, which could induce an opportunity and/or social motivation component.

The results from this review revealed that providing immediate feedback, and/or including a capability component (i.e., individualized exercises) in the exergaming training could potentially have a vital role in enhancing the effect of the exergames on TUG performance. Importantly, caution should be taken when interpreting the meta-analysis concerning the influence of motivation and capability components on exergame outcomes, because this analysis only included one study (51).

### Balance meta-analysis

Results of the meta-analyses indicated favorable outcomes for the exergaming intervention group compared to the control group for all balance measures. Statistically significant effects were found for the BBS, FRT, and PPA measures indicating a significant positive effect of exergame interventions on multiple balance measures. However, these findings should be interpreted with caution with regards to their clinical significance due to the rather small sample sizes. Nevertheless, the magnitude of effects sizes ranged from 0.35 to 0.96, suggesting medium to large effect sizes for these aforementioned tests. Although the Minimal Clinically Important Difference (MCID) of these tasks did not fall within the confidence interval (CI) ranges (87), we could argue that the changes for these tests might be consistent with clinical significance. The MCID for the BBS is determined to be 3 points [(88) however, (89) did not provide any MCID value for the BBS due to its low area under the curve value]. When looking at the baseline vs. follow-up scores for the exergaming groups, there was an improvement in the intervention group of 3.7 points vs. 1.9 points in the control group (44), an improvement of 1.5 points vs. a decrement of 0.06 points for (48), and 3.4 points improvement in the intervention groups vs. 0.93 points in the control group (43). Nevertheless, the rather large CI in the positive range, could indicate that the intervention might be beneficial, but larger sample sizes are needed (87).

The MCD_95_ of the FRT test was determined as 4–11 cm (90). For subacute stroke patients 6.79 cm has been set (91), and 7.32 cm for Parkinson's Disease patients (92). Lee et al. (48) reported a decrement of 0.15 cm in the control group vs. an increment of 4.27 cm in the intervention group. Chen et al. (44) intervention group showed an improvement of 3.80, while the control group only showed 0.50 of increment. The intervention group improved with 4.52 cm, while the control group improved only with 0.44 cm in (52). The test range for the PPA lies between 0 and 3 points, with three indicating a high risk of falling (93). More improvement was seen in interventions groups compared to controls groups among all studies: 0.21 vs. 0.16 (45), 0.20 (KIN intervention = Microsoft Kinect exergames) and 0.50 (SMT intervention = step mat training) vs. 0.20 (46), 0.65 vs. −0.22 (50), and 0.60 vs. −0.01 (53).

Despite this caveat, these findings are in line with recent systematic reviews indicating that exergames induce positive changes in balance function in older adults without neurological disorders (94–96). Pacheco et al. (94) quantitative synthesis showed significant improvements in the BBS (MD = 2.15, 95% CI = 1.77 to 2.56, *p* = 0.0001, *I*^2^ = 96%). Chan et al. (89) concluded that exergames did not significantly improve balance when they examined the effect of different types of exergame interventions on the BBS test (SMD = 0.18, *z* = 0.53, *p* = 0.60, *I*^2^ =71%, Chi^2^ = 6.86). However, in general, they found that exergames reduced the proportion of older adults who fall in the intervention group vs. those in the control groups. The systematic literature review of Choi et al. also suggests that the majority of exergames improve fall prevention, but that it remains inconclusive whether exergames are superior to conventional physical therapy (76).

Favorable outcomes were observed in the exergaming group for muscle strength (STS test), but this did not reach statistical significance. MCIDs were identified as 2.3 s for the STS test (97). While the study by Lee et al. (48) indicated that an exergame intervention had a significant improvement on muscle strength, and that the experimental group showed greater improvements compared to the control group (difference of 4.32 s for the intervention group compared to 0.44 s in the control group); the study of Gschwind et al. (46) reported that only the SMT intervention group showed a clear significant improvement for the STS test (1.70 s). Surprisingly, a small non-significant effect was also found for the TUG test, since almost all intervention groups showed higher improvements compared to the control groups. TUG improvements in intervention groups vs. controls groups were: 1.80 vs. 0.60 s (44), 0.20 vs. 1.00 s (45), 0.40 s (KIN) and 0.00 s (SMT) vs. −0.20 s (46), 1.60 vs. 0.03 s (48), 4.20 vs. 2.30 s (49), 0.67 vs. 0.13 s (50), 0.50 vs. 0.50 s (53), and 3.25 vs. 1.3 s (43). However, the MCID for the TUG test has been determined as 3.40 s (98, 99). In contrast to this result, Pacheco et al. (94) showed significant improvements for this test (MD = −2.48, 95% CI = −3.83 to −1.12, *p* = 0.0003; *I*^2^ = 0%). Exergaming intervention indicated also a moderate effect on TUG performance in the study of Fang et al. (Hedges' *g* = 0.36, 95% CI = 0.26 to 1.30, *p* < 0.001, *I*^2^ = 0%) (95).

Overall, exergames have an effect on a number of balance measures, but this effect is not very large and does not reach what is normally thought as clinically significant. In addition, rather basic balance measures were included in the meta-analyses. Future studies might consider the need to incorporate outcomes for fear of falling, balance confidence, changes in physical activity, dynamic balance and gait measures, such as the Functional Gait Assessment or Mini-BEST, to have a more comprehensive understanding of the benefit of exergames. On the other hand, when looking more closely at the type of exergames used for balance exercises, the X-box Kinect and Nintendo Wii Fit seemed to induce more positive effects than the other exergames. The possible positive influence of exergames on balance performance and the preferable type of exergames to do so should therefore be further examined.

### Cognitive meta-analysis

The results of the meta-analyses show a statistically significant improvement for ANT orienting and MoCA performance, but only a general trend (not statistically significant, small effect sizes) toward improvement of cognitive performance for ANT alerting, ANT executive function, DSB, TMT-A, and TMT-B scores, induced by exergaming intervention. In general, smaller effect sizes were observed for cognition (0.05–0.59) than for balance and physical measures. ANT, DSB, and TMT scores are unlikely to reach clinical significance due to point estimates closer to zero, uncertainty indicated by the large CI intervals, and MCIDs not falling into the CI ranges [not determined for the ANT and TMT yet; 0.45 (1_S.E.M._) to 0.88 (1.96_S.E.M._) for the DSB test (100)]. DSB scores showed smaller improvements compared to their baseline score in the intervention groups, while no improvement was seen for the control groups: 0.10 vs. −0.20 (45), and 0.30 (KIN) and 0.10 (SMT) vs. 0.00 (46). ANT orienting reaction times differences varied from −7.00 ms (KIN), 3.00 ms (SMT), and −13.00 ms (control) in (46), to 0.40 ms (intervention) vs. −7.50 ms (control) in (45). Regarding TMT scores, all three studies included showed post-test improvements, however not significant: for TMT-A, the intervention group improved with 5.5 s (57), 5 s (54), and 2.4 s (45) compared to the control group with 5.8 s (57), 4.5 s (54), and 4.7 s (45). For TMT-B, the intervention group improved with 15.5 s (57), 19 s (54), and 13.9 s (45) vs. 20 s (57), 7.5 s (54), and 19.9 s (45) for the control group.

The MCID for the MoCA is around 2–3 points (101–103)—which does not fall into its CI range—however, the improvement in MoCA performance might indicate clinical significance because of the summary point estimate further from zero, a more positive effect represented by point estimates relatively further from zero, the rather shorter CI intervals, and the more pronounced statistical significance (*p* = 0.01). Participants in the intervention groups of Phirom et al. (50) and Park and Yim (55) demonstrated a significant improvement in MoCA scores compared to their control groups (1.79 vs. −1.35, and 2.06 vs. −1.11, respectively). For Eggenberger et al. (57) only a significant difference between the two groups was found pre-test (for post-test differences of 1.31 vs. 1.07 were found). In line with our results, the meta-analysis of Soares et al. also showed a statistically significant difference between groups for MoCA scores (MD = −1.22, 95% CI: −2.24 to −0.20, *p* = 0.019) (104).

Gschwind et al. conducted a study that comprised two different exergame interventions, which had different outcomes (46). The SMT exergame training showed significant positive pre-post treatment effects for cognitive function performance, while the KIN training would have greater effects on strength and balance (46). On the other hand, X-box Kinect and Kayak 3D exergames improved the MoCA scores significantly. The iStoppFalls used in the study of Gschwind et al. (45) showed rather little improvement for the ANT orienting reaction time. These findings suggest that the type of exergame intervention can have a significant impact on specific outcome measures and the target of the intervention should be carefully considered. Furthermore, Gschwind et al. (45) post-hoc analysis revealed a significantly larger effect in favor of the high-adherence group compared to the control group for executive functioning. They suggest that the duration of the exergame training might play a role, as participants who practiced the exergames for over 90 min per week could improve executive functioning compared to participants who practiced less (45).

In the systematic review of Stojan and Voelcker-Rehage (23) almost all studies reported positive effects of exergame training on cognitive functioning. However, no consistent results could be reported for individual cognitive domains (23). In the literature review of Piech et al., most studies showed that exergames significantly improved various cognitive functions among elderly (22).

Since the exergames used in the studies of these meta-analyses were primarily interested in balance improvement, the cognitive tests used might not have been optimal in terms of detecting cognitive benefits. Exergames which are designed for cognitive improvement might thus result in greater achievements.

### Limitations

There were a number of limitations in the present study. First, the number of studies identified in this review was limited, thereby resulting in small sample sizes in most meta-analyses conducted. There was also marked heterogeneity across studies, with a variety of tools, methods and evaluation strategies used by different studies. This heterogeneity, particularly regarding outcome measures, precluded quantitative meta-analysis for some outcomes. Another limitation of the included studies was lack of long-term follow-up and lack of blinding of interventions or masking of outcome collection. Furthermore, some studies lacked active control intervention, and instead the control groups were asked to perform their usual activity and/or exercises during the trial period. The effectiveness of exergaming may therefore not be completely accurate as the control group can either increase the level of exercises or not perform any exercise regularly. In addition, we did not include languages other than English in our search. In the quantitative meta-analysis of the COM-B model subcomponents, we found that while motivation alone, and capability plus motivation, were associated with improvements in the effectiveness of the intervention, the combination of capability, opportunity and motivation elements in an intervention were not associated with statistically significant changes in effectiveness (Figure 2). We think that this rather counterintuitive finding may be linked to limitations in the evidence that we identified. Whilst we found eighteen eligible studies from which we could extract data on COM-B subcomponents (Table 2), only eight of those presented information on TUG performance and were therefore suitable for the quantitative meta-analysis. Each subcomponent analyses depended on a subset of these eight studies, and in our view should therefore be taken as preliminary findings, pending confirmation in larger samples. Finally, it should be noted that instead of using COM-B to classify components targeting behavior change, a more thorough method could have been adapted at the level of behavior change technique (BCT) for example.

## Conclusion

Motivational factors seem to have an impact on the results of the exergame intervention at least for balance (TUG) performance as examined in this study. Especially motivation components (like providing feedback) and capability components (like individualized exercises) appear to influence the general outcome of the exergame training. Motivational factors could thus be important to consider while setting-up an exergame intervention program for healthy elderly.

This systematic review and meta-analysis suggests that exergame intervention appears to be a promising training method in comparison to traditional exercise training, with positive changes in balance and cognitive performance. However, caution should be taken when drawing conclusions because of heterogeneity in technologies, protocols, sample sizes and outcome evaluation across studies included in the systematic review. Exergame effects were modest to moderate so they may not be sufficient on their own to improve fall risk and cognitive outcomes to a clinically significant degree, but they might supplement traditional physical exercises, or be implemented as part of a multicomponent rehabilitation.

## Data Availability Statement

The original contributions presented in the study are included in the article/Supplementary material, further inquiries can be directed to the corresponding author/s.

## Author contributions

MP and D-EB conceptualized and designed the study and double-rated included articles. MB and YJ collected, organized the data, reviewed the included articles, and conducted the analyses. MB drafted the initial manuscript. MP, SG, and D-EB critically reviewed and revised the manuscript. D-EB coordinated and supervised data collection. All authors read and approved the final manuscript.

## Funding

This work was supported by the European Commission Horizon 2020 grant to D-EB, MP, and SG (grant number 769574).

## Conflict of interest

The authors declare that the research was conducted in the absence of any commercial or financial relationships that could be construed as a potential conflict of interest.

## Publisher's note

All claims expressed in this article are solely those of the authors and do not necessarily represent those of their affiliated organizations, or those of the publisher, the editors and the reviewers. Any product that may be evaluated in this article, or claim that may be made by its manufacturer, is not guaranteed or endorsed by the publisher.
