# Supplementary material for: The role of motivation factors in exergame interventions for fall prevention in older adults: A systematic review and meta-analysis
Source: Front Neurol. 2022 Aug 5;13:903673. doi: 10.3389/fneur.2022.903673 (PMC9388774; doi:10.3389/fneur.2022.903673)
Supplement: Supplementary file 1 [file Table_1.docx]

Supplementary Material.

# Supplementary Table

Table 1. Details of the Selected Studies

| Study | Technology  Used | COM-B | | | Sample | Intervention | Duration | Outcome |
| --- | --- | --- | --- | --- | --- | --- | --- | --- |
|  |  | Cap | Opp | Mot |  |  |  |  |
| Adcock et  al., 2020 | Active@Home Exergame | 🗸 | 🗸 | 🗸 | Healthy and independently living older adults  N=31 (73.9 ± 6.4)  E: N=15  C: N=16 | RCT, two arms  E: Active@Home exergame [(1) Tai Chi-inspired exercises, (2) dancing and (3) step-based cognitive games]  C: Normal daily living | 30-40 min X 3 times a week X 16 weeks  Total: 1440-1920 min (24-32Hr) | STW, DTW, gait analysis (Physilog®5), SPPB, SFT, VST, TMT, WMS-R, MRI |
| Chao et al., 2015 | Wii Fit exergames | 🗸 | 🗸 | 🗸 | Elderly (> 65 y/o) recruited from two assisted living facilities  N=32  E: N=16 (86.63 ± 4.18)  C: N=16 (83.75 ± 8.04) | Quasi-experimental pre/post-test design  Intervention group: SAHA program = motivational intervention + Wii fit exergames  Control group: health education | E: 30 min twice a week for 4 weeks  Total: 240 min (4 Hr)  C: 30 min once a week for 4 weeks  Total: 120 min (2 Hr) | BBS, TUG, Six-minute walk test, GDS, FES, SEE, SF-8TM Health Survey |
| Chen et al., 2020 | X-box Kinect (AR assisted Tai-Chi) |  |  | 🗸 | Community-dwelling adults  N=28  E: N=14 (72.2 ± 2.8)  C: N=14 (75.1 ± 5.5) | Prospective randomized trial, two arms  E: sTC: AR Tai-Chi Training system  C: tTC: Traditional Tai-Chi Training | 30 min X 3 times a week X 8 weeks  Total: 720 min (12Hr) | BBS, TUG, FRT, muscle strength lower extremities |
| Cho et al., 2014 | Nintendo Wii Fit |  |  | 🗸 | Healthy elderly people  N=32  E: N=17 (A=73.1±1.1)  C: N=15 (A=71.7±1.2) | RCT, two arms  E: Wii, balance board and controller  Playing balance games (Ski slalom, table tile, and balance bubble)  C: No intervention | 30 min X 3 times X 8 weeks  Total: 720 min (12 Hr) | Romberg test |
| Eggenberger et al., 2015 | Dancing VR video games | 🗸 | 🗸 | 🗸 | Independently living or at residence facilities older adults (> 70 y/o)  N=71 (6-month test)  DANCE: N=24 (77.3 ±6.3)  MEMORY: N=22 (78.5 ± 5.1)  PHYS: N=25 (80.8 ± 4.7) | RCT  1) virtual reality video game dancing (DANCE)  2) treadmill walking with simultaneous verbal memory training (MEMORY)  3) treadmill walking (PHYS) | Two sessions X 1-hour training sessions per week over 6 months (26 weeks) were applied (1 year follow up also)  60 min X 2 times per week for 24 weeks  Total: 28800 Min (480 Hr) | MMSE, gait performance data, SPPB, Chair-rises, GDS, FES-I, 6-minute walk test |
| Eggenberger et al., 2016 | DANCE = Two Impact Dance Platforms | 🗸 | 🗸 | 🗸 | Elderly (> 65 y/o) living independently or at senior residence facilities  N=42 pre-test  N=33 post-test  E DANCE: N=19 (72.8 ± 5.9)  C BALANCE: N=14 (77.8 ± 7.4) | RCT  Intervention group: DANCE = interactive video game dancing (exergame), as a simultaneous cognitive-motor training  Control group: BALANCE = 20 min conventional balance training and 10 min stretching in each session | 30min X 3 times per week for 8 weeks  Total: 720 min (12Hr) | FES-I, SPPB, TMT-B, Stroop World-Color Interference task, Executive Control task, MoCA, TMT-A. 4-meter walk, 5 chair-rises, extended balance, GDS |
| Gschwind, Eichberg, et al., 2015 | iStoppFalls | 🗸 | 🗸 | 🗸 | Community-dwelling elderly people  N=153 (A=74.7 ± 6.3)  E: N=78 (A=74.7 ± 6.7)  C: N=75 (A=74.7 ± 6.0) | International, multicentre, single-blinded RCT, two arms  E: Personal computer, Google TV set top box, Microsoft Kinect, Senior Mobility Monitor and a Nexus 7 Android tablet  Playing balance and muscle strength exercises games  C: Habitual exercise | 180 min X 16 weeks  Total: 2880 min (48 Hr) | PPA, SPPB, TUG, TMT, VST, DSC, ANT, DSB, EQ-5D, PHQ-9, WHODAS 2.0 |
| Gschwind, Schoene, et al., 2015 | X-box Kinect and Step Mat Training | 🗸 | 🗸 | 🗸 | Community-dwelling elderly people  N= 124 (A=80.8 ±6.6)  E1: N=24 (A=80.1 ± 6.3)  E2: N= 39 (A=82.5 ± 7.0)  C: N=61 (A= 80.2 ± 6.5) | RCT, multiple arms  E1: Kinect  Playing three balance games and five strength exercises  E2: Step Mat Training  Cognitive functions and stepping  C: No intervention | E1: 180 min X 16 weeks  Total: 2880 min (48 Hr)  E2: 20 min X 3 times X 16 weeks.  Total: 960 min (16 Hr) | PPA, TUG, STS, ANT, VST, DSB |
| Katajapuu et al., 2017 | X-box Kinect |  | 🗸 | 🗸 | Community-dwelling elderly people  N=30 (A=71.34 ± 6.62)  E: 10 (A=71)  C1: 10 (A=72)  C2: 10 (A=71.5) | RCT, multiple arms  E: Microsoft Kinect  Playing the exergames  (Skiing, hiking, Pikkuli, China Town Race and RecReha Game)  C1: Physiotherapy exercises  C2: No intervention | 50 min X 2 times X 6 weeks.  Total: 600 min (10 Hr) | BBS, SPPB, Handgrip strength |
| Lee et al., 2017 | Nintendo Wii Fit | 🗸 | 🗸 | 🗸 | Community-dwelling elderly people  N=40 (A=75.96± 4.72)  E: N=21 (A=76.15 ± 4.55)  C: N=19 (A=75.71 ± 4.91) | RCT, two arms  E: Wii, balance board, joystick and polarized glasses  Playing Wii Fit with 3D Video Games (Jogging, Swordplay, Ski jump, Hula-hoop, Tennis and Step dance) and fall prevention  C: No intervention (only fall prevention education) | 60 min X 2 times X 6 weeks  Total: 720 min (12Hr) | APS, BBS, FRT, MLS, OLS, STS, TUG, VM |
| Li et al., 2020 | VR Motion  video game |  | 🗸 | 🗸 | Healthy older adults  N=20 (73.1 ± 7.38)  E: N=10 (73.8 ± 7.35)  C: N=10 (72.4 ± 7.75) | Pseudo-randomization, two arms  E: Motion video game training (Whac-A-Mole: Shape task and animal task)  C: No intervention | 45 min X 3 times a week X 4 weeks  Total: 540 min (9Hr) | n-back, SPM, ANT, OLS, PANAS, IMI, PENS |
| Park et al., 2015 | Nintendo Wii fit |  |  | 🗸 | Community-dwelling elderly people  N=24 (A=65.83 ±8.0)  E: N=12 (A=66.5±8.1)  C:N=12 (A=65.2±7.9) | RCT, two arms  E: Playing Wii Fit balance exercise Game (Soccer Heading, Snowboard Slalom, and Table Tilt)  C: Ball exercise | 30 min X 3 times X 8 weeks.  Total: 720min (12Hr) | Sway, TUG |
| Park & Yim et al., 2016 | Exergame (Kayak 3D) | 🗸 |  | 🗸 | Community-dwelling elderly people.  N=72 (A=73.56 ± 2.93)  E: N=36 (A= 72.97 ± 2.98)  C: N=36 (A= 74.11 ± 2.88) | RCT, two arms  E: Playing 3D virtual reality kayak program and conventional exercise  C: Conventional exercise | 50 min X 2 times X 6 weeks.  Total: 600 min (10Hr) | MoCA, ACT, Standing and Sitting Balance test, Grip strength |
| Phirom et al., 2020 | X-box Kinect |  |  | 🗸 | Community-dwelling older adults  N=39 (69.81 ± 3.78)  E: N=19  C: N=20 | Pseudo-randomized assessor-blind controlled trial, two arms  E: X-box ox Kinect (Fruits hunter, where am I?, Whack a mole, Sky fall, and Crossing poison river)  C: Educational material covering cognitive enhancement and fall prevention strategies | 60 min X 3 times a week X 12 weeks  Total: 2160 min (36Hr) | PPA, TUG, MoCA |
| Sadeghi et al., 2017 | Xbox Kinect | 🗸 |  | 🗸 | Elderly men (> 65 y/o)  N=30 (73.13 ± 7.04 years)  E: N=15  C: N=15 | Single-blinded RCT  Intervention group: Your Shape fitness + Sport Kinect game package  Control group: daily activities | 40 min 3 X a week for 8 weeks  Total: 960 min (16Hr) | Knee proprioception |
| Sadeghi et al., 2021 | X-box Kinect (VR) | 🗸 |  | 🗸 | Community-dwelling older men  N=64 (71.8 ± 6.09)  E1: BT: N=14 (70.4 ± 4.3)  E2: VR: N=15 (74.1 ± 7.0)  E3: MIX: N=14 (70.5 ± 5.1)  C: N=15 (72.2 ± 7.2) | Single-blinded RCT, multiple arms  E1: BT: Balance training  E2: VR: 3 sports games (The Light Race mini-game from the Your Shape fitness package, The target kick and Goalkeeper mini-games).  E3: Mix of BT and VR exercise  C: No intervention, daily life activities | 40 min X 3 times a week X 8 weeks  Total: 960 min (16Hr) | Isokinetic quadriceps and hamstrings strength, TUG, OLS on firm and foam surfaces, tandem stance, 10m walk test |
| Sato et al., 2015 | X-box Kinect |  |  | 🗸 | Healthy elderly people  N=54 (A=69.33 ±5.4)  E: N=28 (A=70.07 ± 5.35)  C:N=26 (A=68.50 ± 5.47) | RCT, two arms  E: Kinect with 3D  Playing the exergames: Apple game, Tightrope standing game, Balloon popping game and One-leg standing game  C: No intervention | 40-60 min X 2-3 times a week (up to 24 times)  Total: 960-1440 min (16-24Hr) | BBS, FRT, CS-30 test |
| Schoene et al., 2013 | Dance Dance Revolution (DDR) | 🗸 | 🗸 | 🗸 | Older adults from residents of independent-living or retirement village  N=32 (77.98 ± 4.5)  E: N=15 (A=77.5 ± 4.5)  C: N=17 (A=78.4 ± 4.5) | Single Blinded RCT, two arms  E: DDR game  Playing step game  C: No intervention (usual activities) | 15-20 min X 2-3 times per week X 8 weeks  Total: 240-480 min (4-8Hr) | CSRT, PPA, TUG, Sway, AST, 5STS, TMT, FES |
| A: Age, ACT: arm curl test, ANT: Attention Network Test, APS: Anterior to Posterior Sway, AST: Alternate Step Test, BBS: Berg Balance Scale, C: Control group, Cap: Capability, CS: 30-second chair stand test, CSRT: Choice Stepping Reaction Time, CST: Chair Stand Test, D: Dance Dance Revolution, DSB: Digit Span Backward, DTW: Dual-Task Walking, E: Experimental Group, EQ-5D: European Quality of Life 5 Dimensions, FES: iconographical fall efficacy scale, FFT: Foot Tapping Test, FRT: Functional Reach Test, FRQ: Fall Risk Questionnaire, FSST: Four Square Step Test, GDS: Geriatric Depression Scale, IMI: Intrinsic Motivation Inventory, MET: Melbourne edge test, MLS: Medial to Lateral Sway, MoCA: Montreal Cognitive Assessment, Mot: Motivation, N: Number of participants, n-back: Adaptive n-Back Task, MRI: Magnetic Resonance Imaging, NR: Not Reported, OLS: One-Leg Stance, Opp: Opportunity, PANAS: Positive Affect Negative Affect Schedule, PAR-Q: Physical Activity Readiness Questionnaire, PENS: PHQ-9: The 9 item Patient Health Questionnaire, PPA: Physiological Profile Assessment, RCF: Rey’s Complex Figure, RCT: Randomized Control Trial, SFT: Senior Fitness Test, SPM: Raven’s Standard Progressive Matrices, SPPB: Short Physical Performance Battery, STS: Sit-to Stand Test, STW: Single-Task Walking, TMT: Trail Making Test, TUG: Time Up and Go, VM: Velocity Moment, VRT: Virtual Reality Training, VST: Victoria Stroop Test, WHODAS: World Health Organization Disability Assessment Schedule, WMS-R: Wechsler Memory Scale-Revised, YPAS: Yale Physical Activity Survey, 5STS: Five Times Sit To Stand. | | | | | | | | |
